# Walking Together: Cross-Protection, Genome Conservation, and the Replication Machinery of *Citrus tristeza virus*

**DOI:** 10.3390/v12121353

**Published:** 2020-11-26

**Authors:** Svetlana Y. Folimonova, Diann Achor, Moshe Bar-Joseph

**Affiliations:** 1 Department of Plant Pathology, University of Florida, Gainesville, FL 32611, USA; 2 Plant Molecular and Cellular Biology Program, University of Florida, Gainesville, FL 32611, USA; 3 Citrus Research and Education Center, University of Florida, Lake Alfred, FL 33850, USA; dsar@ufl.edu; 4 The S. Tolkowsky Laboratory, Department of Plant Pathology, The Volcani Center, Agricultural Research Organization, Bet Dagan 7505101, Israel

**Keywords:** RNA virus, closterovirus, *Citrus tristeza virus*, cross-protection, close protection, superinfection exclusion, virus population collapse, quasispecies divergence, stem pitting, genome conservation

## Abstract

“Cross-protection”, a nearly 100 years-old virological term, is suggested to be changed to “close protection”. Evidence for the need of such change has accumulated over the past six decades from the laboratory experiments and field tests conducted by plant pathologists and plant virologists working with different plant viruses, and, in particular, from research on *Citrus tristeza virus* (CTV). A direct confirmation of such close protection came with the finding that “pre-immunization” of citrus plants with the variants of the T36 strain of CTV but not with variants of other virus strains was providing protection against a fluorescent protein-tagged T36-based recombinant virus variant. Under natural conditions close protection is functional and is closely associated both with the conservation of the CTV genome sequence and prevention of superinfection by closely similar isolates. It is suggested that the mechanism is primarily directed to prevent the danger of virus population collapse that could be expected to result through quasispecies divergence of large RNA genomes of the CTV variants continuously replicating within long-living and highly voluminous fruit trees. This review article provides an overview of the CTV cross-protection research, along with a discussion of the phenomenon in the context of the CTV biology and genetics.

## 1. Brief History of Cross-Protection

The variety and abundance of non-cultivated wild plants as well as the large numbers of cultivated crop plants continue to survive despite being continuously exposed to numerous plant pathogens, including viruses. Although plants lack systems which provide humoral immunity, they are able to successfully confront a wide range of disease agents, primarily, by using many different constitutive and acquired resistance strategies based on either single or multiple genetic components that interact and evade fatal interactions of pathogens and host plants. Additionally, the outcome of a pathogen infection often depends on pre-existing community of microorganisms in the same host, which may engage in the synergistic or antagonistic interactions and enhance or ameliorate the impact of the pathogen-induced disease. One of the outstanding examples of the latter scenario is viral cross-protection. The phenomenon essentially depicts a situation in which host plants previously infected with a symptomless or mild variant of a virus species are protected from disease imposed by a subsequent encounter with a closely related severe variant of the virus. Unlike the more commonly recognized plant protection systems based solely on host plant genetic resources, cross-protection is apparently the result of interactions between the genetic traits of the protecting and challenging viruses. This generalization may, however, be questioned. Past practical experience has suggested that certain mild isolates of *Citrus tristeza virus* (CTV) that provided satisfactory field protection against severe stem pitting isolates of the virus for “Marsh” seedless grapefruit (*Citrus paradisi* MacFadyen) failed to provide similar protection when applied under similar conditions to the “Star Ruby” variety of grapefruit plants [1].

The phenomenon of cross-protection was first observed almost one hundred years ago by McKinney [2]. He found that infection of a tobacco plant by a phenotypic variant of *Tobacco mosaic virus* (TMV) prevented its subsequent infection by a TMV variant with a different phenotype. Salaman [3] extended this finding to practical levels by showing that tobacco plants infected with a mild isolate of *Potato virus X* were protected from the following infection by other more aggressive isolates of the same virus, although they continued to be susceptible to infection by two unrelated viruses, TMV and *Potato virus Y*. These results continued to encourage plant virologists in different geographical locations to explore the use of such a protection strategy for practical needs. Interest intensified particularly as a result of outbreaks of emerging viral diseases for which breeders were lacking genetic resources or the process of resistance breeding was difficult and/or lengthy. However, as indicated by Fulton [4], the majority of these attempts to control virus diseases of fruit trees, with a few exceptions (e.g., papaya; [5]), were generally disappointing.

## 2. Application of Cross-Protection against CTV

Among the outstanding examples of controlling a serious plant viral disease via cross-protection is the wide-scale use of mild CTV isolates to control the manifestation of devastating stem pitting disease, which could be induced by some aggressive virus isolates. The typical quick decline—“tristeza”-symptoms of citrus trees grafted on the sour orange (*C. aurantium* L.) rootstock could be effectively controlled by changing to citrus rootstocks or citrus relatives conferring tolerance or resistance to CTV. However, the stem pitting syndrome of CTV affects the citrus scion segments, regardless of the rootstock reaction to the virus [6,7]. The manifestation of CTV-induced stem pitting is especially grave in certain citrus genotypes. These include the Brazilian sweet orange (*C. sinensis* (L.) Osbeck) variety–cv. Pera, white and red skin grapefruits, and Oroblanco, a hybrid of grapefruit and acid-less pummelo (*C. grandis* Osbeck x *C. paradisi* MacFadyen). Trees of sensitive citrus scion varieties, when infected with stem pitting CTV isolates, are severely stunted and perform poorly, producing fruits of small size and low quality. Indeed, the stem pitting disease of citrus trees, rather than the more commonly known tristeza decline, is considered to be the most significant and difficult to control manifestation of CTV.

**Figure 1:**
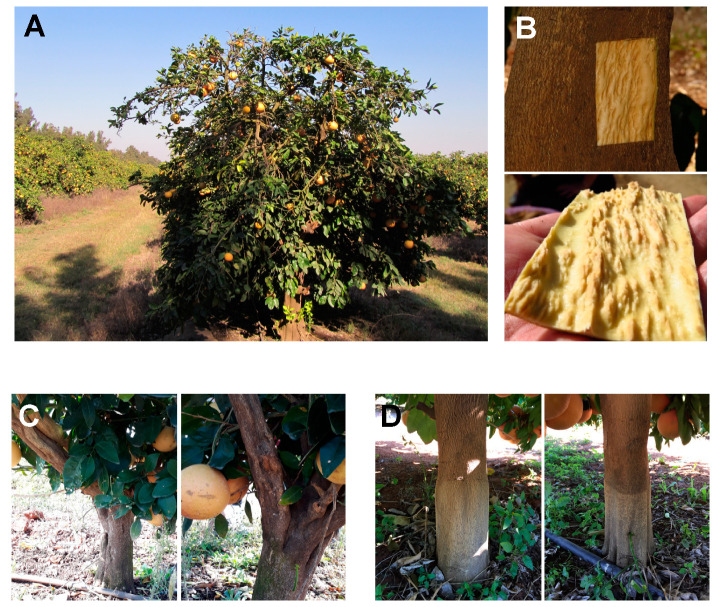
CTV-induced stem pitting. (**A**) A field citrus tree showing stem pitting. (**B**) Pits on the scion part of a trunk of a citrus tree affected by the CTV-induced stem pitting along with the corresponding piece of bark peeled from the tree trunk. (**C**) Trunks and lateral branches of Star Ruby grapefruit trees affected by stem pitting. (**D**) Trunks of cross-protected Star Ruby grapefruit trees. Images were kindly provided by Dr. Glynnis Cook.

During the past seventy years, citriculturists have experimented with selection of mild CTV isolates for protecting and saving the citrus industries in Australia [8,9,10,11], Pera orange production in Brazil [12,13,14], white Marsh and red Star Ruby grapefruit in South Africa [15,16], and Peru [17,18] (Figure 1). All these successful citrus-growing industries were saved and continue to function due to cross-protection.

## 3. Terminology

Despite marked differences in the defense strategies of plants and animals against invading pathogenic viruses, the observation of disease elimination mediated by the cross-protection phenomenon led Costa and Müller [12] to popularize the term “preimmunization”. They first introduced the term when reporting on certain selected mild CTV isolates during an attempt to revive the Brazilian citrus industry following the devastating damage caused by the local stem pitting CTV isolates. Other citrus industries, including the South African, where grapefruit orchards are totally dependent on cross-protection, also adapted the Brazilian term. Other names given to the cross-protection phenomenon include antagonism, homologous interference, and superinfection exclusion (SIE); the last used primarily in studies of the protection mechanism.

## 4. Cross-Protection Research: A Personal Recollection

Cross-protection research was, for most of its history, based on the arbitrary selection of mild CTV isolates followed by tests of their effectiveness in providing protection against local isolates. A major change in this situation occurred with the development of an infectious cDNA clone of CTV, allowing analyses of genetic relatedness between the protecting and challenging isolates [19,20,21]. These results, showing that cross-protection is not dependent on CTV isolate symptomatology, but on genetic similarity, were of seminal significance [22]. Researchers now understood that, when numerous attempts at control with perfectly mild isolates were unsuccessful, it was due to the fact that the isolates belonged to different CTV strains. This review article attempts to provide an overview of the authors’ past cross-protection research, along with a discussion of the phenomenon in the context of the CTV biology and genetics.

From 1966, when one of the authors of this article, Moshe Bar-Joseph, enrolled as a PhD student to work on CTV in Israel, until approximately twenty years later, when the major suppression efforts of field cases of CTV infections were discontinued, the subject of cross-protection research had to be confined to glass house conditions. With these limitations, the tests were performed, which demonstrated that some of the local isolates from asymptomatic trees (e.g., ST Miqveh-T and 127000-T) were able to protect sour orange seedlings against the severe seedling yellows symptoms induced by the CTV isolates VT and Mor-T (M. Bar-Joseph, personal communication). Initially, the Miqveh-T mild isolate also showed outstanding protection for sweet orange trees on the sour orange rootstock, which were planted in *Toxoptera citricida-*infested citrus fields at the Nelspruit citrus and subtropical station, South Africa. Later studies, however, discouraged the use of the mild isolate Miqveh-T of the VT strain [23] due to its severe stem pitting on Star Ruby grapefruit [24,25] and Oroblanco pummelo –grapefruit hybrids [25]. Realizing the danger of natural spread of the stem pitting isolates to grapefruit and Oroblanco groves from a large-scale field experiment designed to test the possible protection of a mature Valencia sweet orange grove on the sour orange rootstock, the decision was made to abandon and uproot the testing plot, which was surrounded by large commercial plantings of sensitive grapefruit and Oroblanco genotypes. Indeed, even today, approximately 30 years after halting field experiments, the sensitive groves in the area remain free of stem pitting symptoms. It should be noted, however, that the absence of natural spread of the stem pitting isolates, as described above, could also have been due to the less effective transmission of the mild VT isolates by the local vector *Aphis gossypii.*

## 5. Cross-Protection among CTV Isolates Displaying Differential Transmission by *A. gossypii*

Cross-protection was mostly tested between isolates showing phenotypic differences in symptomatology. In 1978, a Letter to the Editor published in *Phytopathology* [26] reported puzzling observations in the Israeli citrus-growing areas where natural virus spread by local vectors such as *A. gossypii* was only noticed about three decades after the introduction (in the 1920′s) of citrus varieties infected with exotic CTV variants. The spread of the introduced variants was first observed in 1969, with circumstantial evidence suggesting that natural infection and tree decline began at least 2–3 years earlier. The major difference between the originally introduced isolates vs. the isolates recovered from the local spread of the virus was evident. The introduced isolates were non- or poorly transmitted by *A. gossypii,* while isolates collected from recent incidences of natural spread were highly transmissible by different *A. gossypii* colonies. This was an indication of the emergence of a mutant CTV variant(s) well adapted to the local aphid species. Given a large number of CTV virions being produced continuously in the field citrus trees and an abundance of aphids, the emergence of *A. gossypii*-transmissible CTV variants and the shift in the CTV populations would have been expected to occur at least two decades earlier. The long lag period pointed to a mechanism that prevented the timely emergence of CTV variants adapted to transmission by *A. gossyppi*. In order to test a possibility that conservation of the non-transmissible isolate resulted from cross-protection of the parental isolate against a transmissible one, we challenged plants carrying a poorly transmissible isolate ST using a highly transmissible isolate VT. The result was a marked reduction in *A. gossypii* transmission of the recovered virus isolates [26].

It is interesting to note that effective cross-protection was obtained using ST rather than the other, much more poorly transmissible CTV isolates described by Bar-Joseph [26]. Both CTV isolates ST and VT were found years later to belong to the VT strain [23]. These experiments demonstrated that the cross-protection mechanism not only provides protection against the more severe symptoms of the challenging isolate, but that it is also able to prevent the natural spread of the challenging, transmissible CTV isolate by the insect vector.

## 6. Failure of Cross-Protection between CTV Variants of Different Strains

Rosner et al. [27] described cloning of cDNA fragments corresponding to the CTV isolate VT. A follow-up paper [28] addressed an interesting question concerning the genetic relationship between different CTV isolates, which were collected through the suppression program in Israel. The hybridization assays of the extracts from different CTV isolates using a VT-specific probe ‘D1′ demonstrated genetic diversity of the local CTV isolates. The genetic diversity, however, did not correlate with previously noted biological properties of these isolates. Among the isolates that were not recognized by the VT probe was HT. The isolate was from a sweet orange (cv. Valencia) tree grown on the sour orange rootstock showing quick decline that was located at a distance of about 25 km south of the CTV VT-infected orchards. The symptoms of CTV HT on the Mexican lime (*C. aurantifolia*) indicator plants were less aggressive, compared with those of VT. Unlike CTV VT, which showed a strong seedling yellows reaction, the CTV HT infections were apparently symptomless. Instead of the classical cross-protection testing based on prevention of differential symptoms, we used a new approach in which the RNA extracted from the infected plants was hybridized with an isolate-specific cDNA probe. While CTV VT gave a strong hybridization signal, CTV HT was not recognized by the VT D1 probe [28]. The cross-protection experimental procedure included graft inoculation of *C. macrophylla* with the HT isolate, followed by topping plants after 2–3 weeks, and, upon symptom appearance, challenging those by inoculation with the CTV VT-carrying budwood, along with a similarly inoculated group of healthy control plants. The two groups underwent a second topping after about a month and were tested by hybridization, soon after showing virus symptoms. The hybridization results were similar: both groups showed clear and strong hybridization signals with the VT-specific D1 probe due to the presence of CTV VT, including all plants, which were pre-inoculated with CTV HT. We considered these results to be negative and not worth publishing, since it was already well known from the seminal work of Costa and Muller [11] that cross-protection can occur only in the case of some CTV isolates. The summer student who conducted the hybridization tests left the laboratory feeling very frustrated; and we missed the understanding that it was the extensive genetic divergency between VT and HT that led to the cross-protection failure. Approximately ten years later, Cline and his team at the University of Florida sequenced the CTV T36 coat protein gene [29], and, based on their sequence, Mawassi et al. [23] analyzed the sequences of the coat protein genes of the CTV isolates from Israel. These results demonstrated that the local Israeli VT isolate coat protein sequence was almost identical to the coat protein genes of other severe isolates as well as of the isolates from some of the asymptomatic trees. Based on this, we suggested to use the name ‘VT’ for both the isolate and as the name of a group of CTV isolates with closely related sequences, including some with rather different biology, which later became the VT name of a CTV strain.

Interestingly, two of the sequenced CTV isolates, including CTV HT and the Meyer lemon (*C. meyeri*) MT isolate, showed strikingly different genetic composition of their coat protein genes as compared with isolates of the VT strain [23]. In retrospect, this explained both the inability of the VT probe to recognize the CTV HT RNA sample by dot blot hybridization [28] and the discussed above failure of the HT-infected plants to prevent superinfection by CTV VT (Bar-Joseph, unpublished). Unlike the variants of the VT strain, which became widely spread in Israel, especially, after the discontinuation of the CTV suppression program, HT went unnoticed until the published sequence of the coat protein gene of CTV HT [23] was found to be almost identical to that of the Floridian T36 (S. Harper, personal communication). By that time, Folimonova et al. [21] had already showed that cross-protection was only possible between CTV variants belonging to the same strain.

## 7. CTV Genetics

**Figure 2:**
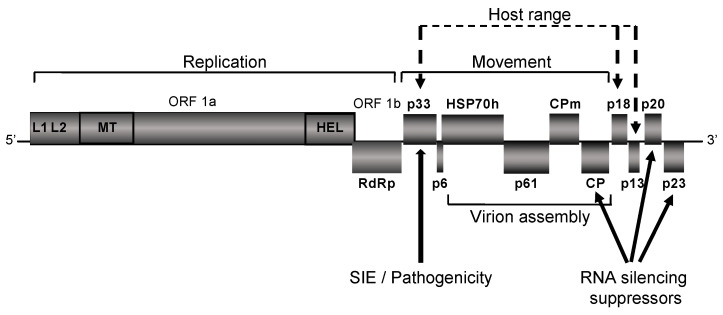
Schematic diagram of the CTV genome organization. The open boxes represent open reading frames (ORFs) and their translation products. L1, L2, papain-like leader protease domains; MT, methyltransferase-like domain; HEL, helicase-like domain; RdRp, an RNA-dependent RNA polymerase; HSP70h, HSP70 homolog; CPm, minor coat protein; CP, major coat protein; SIE, superinfection exclusion.

CTV has several peculiarities, which set it apart not just among the elongated plant viruses, but even within the *Closteroviridae* family itself. The CTV virions (2000 nm × 10–12 nm) and the 19.3 kb single-stranded positive-sense non-segmented RNA genome are among the largest of the RNA viruses [30,31,32,33,34,35,36]. The RNA genome of CTV contains twelve open reading frames (ORFs) [37] (Figure 2) that encode polyproteins required for virus replication (ORFs 1a and 1b); major (CP) and minor (CPm) coat proteins, p65 (HSP70 homolog), and p61, which are involved in assembly of virions [38]; a hydrophobic p6 protein with a proposed role in virus movement [32,39] as well as p20 and p23, which along with CP are suppressors of RNA silencing [40]. The CTV genome also contains a few unique genes such as those of the p33, p13, and p18 proteins, which are disposable for virus infection in susceptible hosts [39,41].

A notable feature of CTV infections is the abundance and variety of the repertoire of subviral genetic elements consisting of different populations of single-stranded and double-stranded RNA molecules. Among those are two relatively short RNA molecules and a long one that comprise the sequences corresponding to the 5′-terminal fragment of the genome of about 650–750 nucleotides in size or to a region encompassing the complete ORFs 1a and 1b, respectively [42,43,44,45]; an array of different in length 3′-terminal subgenomic RNAs, serving for expression of the distal 3′-end genes of the CTV genome along with their negative-sense complementary copies [46,47]; and multiple types of defective RNA molecules consisting of the 5′- and 3′-terminal genomic regions interspaced, in some cases, with variable in size sequences from other CTV genomic parts. Interestingly, none of these CTV defective RNA molecules were showing the phenomenon of defective RNA interference reported to be taking place between defective interfering RNAs and the helper genomes in several other virus systems [48].

CTV stands out from most viruses for unprecedented variability of variants, which have distinctive biological and genetic characteristics. The variants can be classified into at least seven major CTV genotype groups (strains): T3, T30, T36, VT, T68, RB, and HA 16-5 [21,49,50]. Strains represent phylogenetically distinct lineages of CTV, which are defined based upon analysis of the nucleotide sequences of ORF 1a. This genomic region displays highest genetic diversity between CTV variants, with levels of sequence identity ranging between 72.3–90.3% for virus variants from different strains [49,50,51,52,53,54,55]. More conserved 3′-half regions show 89–94.8% identity. Each strain is composed of variants with minor sequence divergence, generally, less than 5% throughout the entire genome. With that, viruses within a strain may have significant variations in symptoms.

A remarkable characteristic of CTV is the slow evolutionary rate, which ranges from 10^−4^ to 10^−5^ nucleotides per site per year, thus, placing CTV in the 10th percentile of the most slowly evolving viruses [56]. Strong genetic conservation was first noted in reports showing high sequence conservation among virus variants with a common origin that were geographically separated for several decades [57,58]. Analysis of the nucleotide changes in the progeny of the infectious clone of CTV T36 propagated in a *C. macrophylla* tree under the greenhouse conditions for seven years revealed only nine nucleotide substitutions, which equals an evolutionary rate of 6.67 × 10^−5^ nucleotide alterations per site per year [50]. The slow mutation rate of the large CTV RNA genome is consistent with reports of a weak negative correlation between RNA virus genome size and evolutionary mutation rate [59,60], which was noted for large animal RNA viruses. Accumulation of degenerated sequences resulting from lethal genetic errors in viruses with large genomes must come with a heavy cost. Indeed, the large 30+ kb single-stranded RNA genomes of coronaviruses encode a 3′–5′ exoribonuclease [61], which provides proofreading repair of the replicating large genomes [62,63].

Another peculiarity of the CTV-plant host interactions is the continuous and repeated failure to obtain durable transgenic resistance of citrus plants using cDNA constructs corresponding to viral genes [64,65] (L. Peña, personal communication). A number of CTV genes, including the CP, p20, and p23 genes as well as noncoding virus genome-derived sequences designed to produce self-complementary transcripts inducing the RNA silencing-based antiviral response have been tested with low success. These observations contrasted to multiple reports for other plant RNA viruses in which transgenic expression of viral coat protein sequences (as well as other additional genes) was shown to effectively protect plants against viral infections [66]. The unsuccessful attempts to generate transgenic resistance to CTV have left cross-protection with appropriate mild virus isolates as the only means to protect commercial citrus varieties from CTV-associated stem pitting. Furthermore, although quick decline could be effectively managed by the use of resistant and/or tolerant rootstocks, finding mild virus isolates that could provide sustained protection against this disease would allow to bring sour orange, a highly adaptable to various soil types and tolerant to the oomycetes-associated root rot diseases rootstock, back into play in many citrus growing regions where it has been abandoned due to the presence of decline-inducing isolates of CTV.

## 8. Understanding the Mechanism of CTV Cross-Protection

Development of an infectious cDNA clone of CTV based on an isolate of the T36 strain followed by engineering a virus variant tagged with a reporter gene (e.g., that of the green fluorescent protein (GFP)) [19,20] were major breakthroughs that took the examination of cross-protection between CTV variants to the next level. The outcome of cross-protection tests was now assessed not by observing suppression of the challenge variant symptoms or a lack of that, but rather by the ability of the GFP-expressing virus to superinfect trees that were pre-infected by virus variants belonging to different strains. This approach unequivocally demonstrated that cross-protection and, specifically, SIE occurs only between variants of same CTV strain. On the other hand, variants of different CTV strains do not exclude each other allowing the challenging virus variant to spread and multiply in trees infected with the preexisting virus [21]. The observations made in that seminal work had several important outcomes. First of all, they provided an explanation of why some earlier cross-protection tests have failed. One of the cross-protection failures (inability of the isolate HT to cross-protect against the VT isolate) was discussed above. Another example comes from a long history of numerous attempts to achieve protection against a decline-causing isolate of the T36 strain by using mild isolates of the T30 strain in Florida (reviewed in [67]). Despite all the efforts during several decades over the second half of the past century, no sustained cross-protection was achieved with such combinations, precluding the use of the sour orange rootstock in the Florida citrus groves. Second, the discovery that CTV variants of different strains do not exclude each other brought an understanding of previously unexplained features of the CTV biology, and, in particular, explained the formation of complex populations made up of variants of different CTV strains in the field citrus trees. Indeed, long-living field citrus trees often harbor complex populations made up of variants of different strains [52,53,54,68,69,70,71]. Lack of exclusion between different strains permits the establishment of multiple heterologous virus variants in the same tree upon repeated aphid-mediated virus introductions under the field conditions. Third, the findings described in Folimonova et al. [21] built a foundation for the strategy of selecting protecting virus variants to control aggressive variants of CTV in the field. That work, however, was conducted using ‘pure-culture’ isolates that contained only a single virus lineage. To model the situation with the field trees, a follow-up study examined SIE in plants pre-infected with several variants of different CTV strains [72]. The experiments showed that exclusion of the challenging virus was triggered by the presence of another variant of the same strain in the primary population and was not affected by co-occurrence of additional heterologous lineages. It is important to note here that, although this research has significantly advanced our knowledge on CTV SIE, many questions related to the factors affecting practical outcomes of field cross-protection remain to be answered. With that, however, cross-protection among CTV isolates can already be termed as “close protection”.

Examination of the CTV SIE mechanism revealed that it is mediated by multiple components. Remarkably, sequences in the 3′-half of the CTV genome did not appear to have any effect on SIE [21]. Hybrid viruses engineered based on a cDNA clone of the T36 variant in which 3′-half regions were substituted with the cognate sequences derived from the genomes of heterologous variants of the T68 or T30 strains retained the capacity to exclude a subsequent infection by the T36 variant, while did not gain the ability to protect against the other donor variants. Moreover, the protection against the secondary homologous virus variant was shown not to be a simple case of host RNA silencing as it has been suggested for other plant viruses [73], but rather function as an active virus-controlled phenomenon [74]. The mechanism mediating exclusion between the variants of the same strain was found to involve a viral non-conserved p33 protein as well as a 5′-terminal region of the virus genome encoding the two short, positive-sense subgenomic RNAs and the two viral leader proteases [75,76]. Deletion of the p33 ORF from the viral genome or a frameshift mutation resulting in a loss of functional p33 completely removed virus ability to exclude superinfection by a closely related variant [75]. Furthermore, alterations in the 5′-terminal region of the CTV genome affected SIE as well [76]. As mentioned above, the latter genomic area shows highest diversity among the CTV variants, so it could be hypothesized that it encodes a factor(s) allowing the primary virus to distinguish between a “self” vs. “non-self”, thus, determining the outcome of the challenging virus infection.

## 9. Cross-Protection, Genome Conservation, and the Replication Machinery of CTV: Are These Properties Related?

It has been accepted that viral RNA polymerases possess low copying fidelity. Calculations of mutation frequencies for a number of RNA viruses supported the values ranging from 10^−3^ to 10^−5^ substitutions per nucleotide copied. It was suggested that, on average, replication of RNA viruses with the genome length between 3 kb and 32 kb results in 0.1–1 mutation introduced per RNA template copied. Some of those may be lethal and impede further replication of a mutant genome. Nevertheless, a continuous input of mutant virus variants would be expected [77]. On the other hand, lineages of CTV maintain high genetic stability over time. As discussed above, a rate of CTV evolution under stable conditions ranges between 10^−4^ and 10^−5^ nucleotides per site per year. Closteroviruses lack the proof-reading exonuclease reported in coronaviruses [32,56,62]. Hence, their large genomes and outstanding genomic stability suggest that they possibly employ some alternative strategies of genome conservation that prevent accumulation of mutants, which potentially could result in population collapse.

SIE, which operates between closely related virus variants, could be a two-function mechanism that (i) regulates genomic stability of CTV by preventing co-replication of newly produced mutated progeny arising from error-prone replication process and (ii) prevents implantation of new variants with closely similar 5′-terminal genomic regions. Indeed, experiments with animal alphaviruses have demonstrated that the phenomenon of SIE could be established within a fraction of an hour after the primary virus enters the cell [78,79]. Furthermore, for a number of RNA viruses, it was shown that the block of a subsequent homologous virus infection occurs at the replication level [78,80,81,82,83,84]. Among those examples is a study on SIE by *Turnip crinkle virus* (TCV), a small (ca. 4 kb) positive-sense RNA virus in the family *Tombusviridae*, which infects cruciferous and some non-cruciferous plants. In this study, Zhang et al. [84] showed that exclusion of a secondary infection with a TCV variant was mediated by the replication protein p28 produced by the primary-infecting virus variant. The authors further speculated that, besides preventing sequential infections by the homologous superinfectors in the cells pre-occupied by the ‘resident’ virus variant, SIE also targets its newly produced progeny, thus, maintaining an optimal error frequency in the synthesized genomes [84,85].

**Figure 3:**
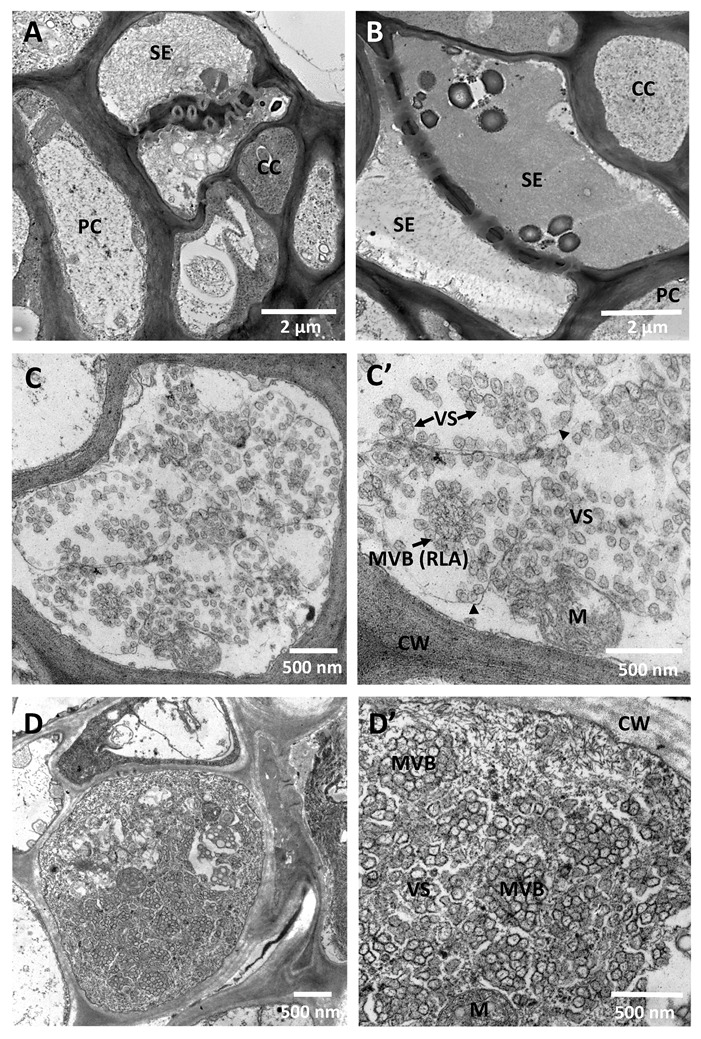

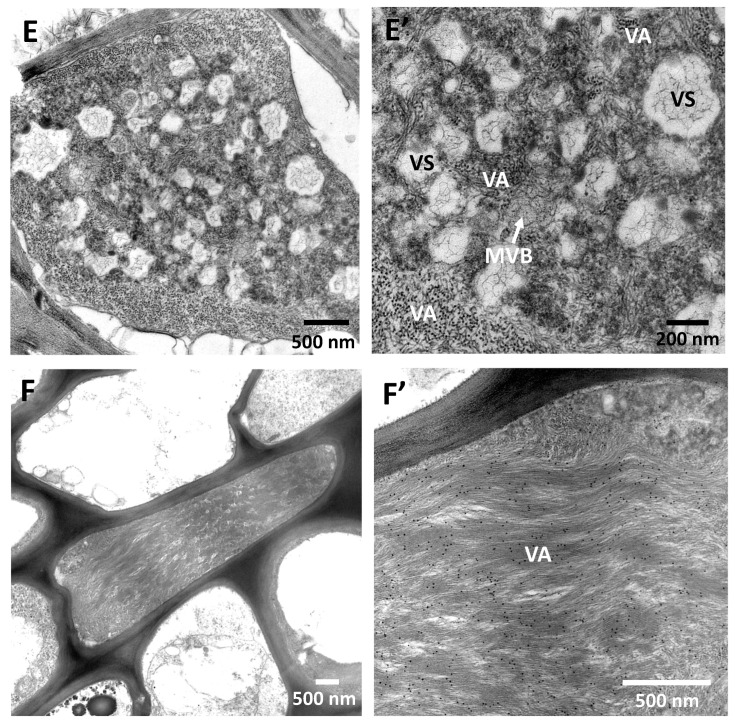
CTV-associated cytopathology. Transmission electron micrographs showing phloem cells in a petiole of a healthy (**A**,**B**) or a CTV-infected (**C**–**F’**) *C. macrophylla* plant. (**C**,**D**,**F**) Virus-infected phloem parenchyma cells at lower magnification. (**C’**,**D’**,**F’**) Areas from the cells shown in (**C**,**D**,**F**) at higher magnification. Viral arrays in (**F**,**F’**) were labeled with a polyclonal CTV-specific antibody used as the primary antibody and a secondary antibody conjugated with 10-nm gold particles as described in Folimonova et al. (2008) [99]. SE, sieve element; CC, companion cell; PC, parenchyma cell; CW, cell wall; M, mitochondrion; VS, vesicles; MVB, multivesicular bodies; RLA, rosette-like arrangement; VA, virion arrays. Size bars are indicated.

All positive-sense RNA viruses remodel cellular membranes to form viral replication organelles [86]. Those structures provide spatial separation of the different steps of the virus cycle, enable local enrichment of the viral replication proteins and required host factors and metabolites as well as shield viral RNA from the cellular nucleases and sensors of the innate immune surveillance [87,88]. Infection with CTV induces formation of ~100 nm double-membrane vesicles and multivesicular bodies, which are presumed to be viral replication organelles (Figure 3). Such vesicular structures were originally described for *Beet yellows closterovirus* (BYV) [89] and then observed for other closteroviruses [30,34]. The CTV membraneous vesicular structures are often seen forming rosette-like arrangements in which the “necks” of the vesicles are positioned towards the electron-dense center (Figure 3C,C’). Those are most abundant in the phloem tissue cells, at early stages of virus infection (Figure 3C–D’). Cells at the late stages of infection usually show vesicles, which undergo degradation and are surrounded by the virion arrays (Figure 3E,E’). Alternatively, those cells could be fully packed with loads of the assembled virions (Figure 3F,F’). Similar to what was shown for *Lettuce infectious yellows virus*, another member of the family *Closteroviridae*, inoculation of plant protoplasts with a replicon of CTV carrying only replication-associated genes (ORFs 1a and 1b) led to active replication of the introduced virus construct and concominant formation of the characteristic double-membrane vesicles and multivesicular bodies ([90], S. Y. Folimonova, unpublished). A role of these virus-induced organelles in the replication of this group of viruses is also supported by the finding that BYV leader protease, methyltransferase, and helicase protein domains colocalize with the vesiculated bodies [91,92], and the formation of the latter structures is triggered by another domain located in the central region of ORF 1a [93]. The containment of the replication machinery of CTV in the enclosed membranous structures may play a role in segregating virus populations. During the advanced stages of the primary virus replication, the exclusion mechanism may block generation of additional replication organelles or prevent progeny genomes or superinfecting viral RNA from entry into pre-existing functional replication complexes. The spatial separation could also serve as a regulatory mechanism permitting sequestration of genetically related superinfecting genomic RNA molecules (or the respective negative strands) by their complementation with the homologous full-length or subgenomic RNA molecules produced by the primary virus allowed to exit the replication organelle. The sequestered RNAs will be removed from a pool of active replicators and exposed to the hostile cytoplasmic environment. Taking into account recent observations from a number of plant viruses showing that replication factories and multivesicular bodies can move from cell to cell through plasmodesmata [94,95,96,97,98], one can also suggest that the enclosure of the primary virus genomes in those structures provides an additional competitive advantage at the level of the intercellular movement.

With CTV, the role of SIE in maintaining the integrity of the founder virus genome is especially important. CTV infects long-living citrus trees in which the virus can persist for the whole tree lifespan that could be more than 50 years. Because of so lengthy infections, the ‘resident’ virus also needs to protect itself from repeated introductions of highly similar variants mediated by vectoring insects. Importantly, even in the most susceptible citrus hosts (i.e., *C. macrophylla*), CTV infects only a proportion (one-third or less) of susceptible cells, leaving the majority of the plant cells free of the virus [21,99]. However, the cells not occupied by the primary virus appear to be effectively protected from the homologous superinfectors as well [21]. This situation suggests that, in case of CTV, SIE operates at two levels—the cellular and the whole-organism levels. It is intriguing that, as we found, CTV SIE relies on multiple viral factors, and the viral p33 protein mediates its non-cell-autonomous phase [76,100]. We, therefore, hypothesize that SIE blocking the target variant(s) replication is initially established in the cells infected by the founding virus, which possibly involves viral factor(s) encoded in the 5′-region of the viral genome. The phenomenon then spreads beyond the infected cells, and this step requires the p33 protein. Thus, CTV takes the SIE-based “self-defense” strategy to the next level, which provides efficient elimination of closely related competitors, whether those are progeny genomes or virus variants introduced from the outside, and creates ideal ecological niche for the “resident” virus.

## 10. Concluding Remarks

“Cross-protection”, a nearly 100 years-old virological term, could be now changed to “close protection”. Evidence for the need of such change accumulated before the genome sequencing era with finding that a mild nitrous acid-induced variant of *Papaya ringspot virus* (PRSV) provided effective protection against severe PRSV isolates only in the geographical areas where the protective mild variant originated and failed in other locations harboring different virus strains [5,101]. The first direct evidence for such close protection was obtained with CTV with the use of a fluorescent protein-tagged recombinant virus variant of the T36 strain, which failed to superinfect plants pre-infected by variants of the same (e.g., T36) strain, while it was able to establish infection in plants carrying variants of other CTV strains. Hence, the protection is only active against closely related virus variants, which is in accordance with the expected necessity of large RNA genomes to protect themselves against the danger of population collapse expected to result through quasispecies divergence of RNA virus genomes continuously replicating within long-living and highly voluminous fruit trees. From a standpoint of terminology, unlike cross-protection, which often is used to describe the outcome of interactions between related viruses revealed by interference with the expression of the secondary virus symptoms (phenotype), close protection primarily delineates the mechanism of virus genotype conservation. From a practical standpoint, close protection offers exciting new possibilities for selecting mild protective CTV variants with close genetic identities to the prevailing pathogenic variants infecting a citrus area. It also points at the danger of superinfection by genetically different variants of novel CTV strains and, therefore, calls for continuous efforts of regulatory agencies to prevent the introduction of new exotic variants of the virus, even into the areas where CTV is already widely present and efficiently controlled by cross-protection.
